# Identification of key genes in late-onset major depressive disorder through a co-expression network module

**DOI:** 10.3389/fgene.2022.1048761

**Published:** 2022-12-06

**Authors:** Ping-An Yao, Hai-Ju Sun, Xiao-Yu Li

**Affiliations:** ^1^ School of Pharmaceutical Sciences, Zhejiang Chinese Medical University, Hangzhou, China; ^2^ Department of Neurobiology and Acupuncture Research, The Third Clinical Medical College, Zhejiang Chinese Medical University, Key Laboratory of Acupuncture and Neurology of Zhejiang Province, Hangzhou, China; ^3^ The Third Affiliated Hospital of Zhejiang Chinese Medical University, Hangzhou, China

**Keywords:** late-onset major depressive disorder, weighted gene co-expression network analysis, differentially expressed genes, immune infiltration, hub gene, receiver operating characteristic

## Abstract

Late-onset major depressive disorder (LOD) increases the risk of disability and suicide in elderly patients. However, the complex pathological mechanism of LOD still remains unclear. We selected 10 LOD patients and 12 healthy control samples from the GSE76826 dataset for statistical analysis. Under the screening criteria, 811 differentially expressed genes (DEGs) were screened. We obtained a total of two most clinically significant modules through the weighted gene co-expression network analysis (WGCNA). Functional analysis of the genes in the most clinically significant modules was performed to explore the potential mechanism of LOD, followed by protein–protein interaction (PPI) analysis and hub gene identification in the core area of the PPI network. Furthermore, we identified immune infiltrating cells using the cell-type identification by estimating relative subsets of RNA transcripts (CIBERSORT) algorithm between healthy subjects and LOD patients with the GSE98793 dataset. Next, six hub genes (*CD27*, *IL7R*, *CXCL1*, *CCR7*, *IGLL5*, and *CD79A*) were obtained by intersecting hub genes with DEGs, followed by verifying the diagnostic accuracy with the receiver operating characteristic curve (ROC). In addition, we constructed the least absolute shrinkage and selection operator (LASSO) regression model for hub gene cross-validation. Finally, we found that *CD27* and *IGLL5* were good diagnostic indicators of LOD, and *CD27* may be the key gene of immune function change in LOD. In conclusion, our research shows that the changes in the immune function may be an important mechanism in the development of LOD, which can provide some guidance for the related research of LOD in the future.

## Introduction

As a common mental disorder with high morbidity, major depressive disorder (MDD) is the main cause of severe disability in patients (Kessler et al., 2003). The age distribution of the MDD onset shows that depression is common during the whole life cycle (Wagner et al., 2020). However, the effective treatment of MDD is currently followed in less than half of the patients (Dowlati et al., 2010). Complex physiological and pathological conditions are the main reason for its refractory state. Late-onset MDD (LOD) usually occurs in patients whose onset age is greater than or equal to 50 years (Miyata et al., 2016). Compared with non-LOD patients, LOD patients have a higher risk of suicide and cognitive and psychosocial disorders (Alexopoulos et al., 1988), which greatly increases the social and family burden. The lack of specificity of diagnostic methods and the decline of body function, such as the decline of the immune system function and the increase of combined diseases, contribute to the intractability of LOD. In most cases, LOD can be accurately identified by the Diagnostic and Statistical Manual of Mental Disorders (DSM). Unfortunately, LOD combined with other psychiatric disorders makes it different (Das-Munshi et al., 2008). Therefore, it is urgent to improve the diagnostic accuracy of LOD patients using physiological and pathological indexes.

The involvement of immune response in the process of MDD has been confirmed, such as immune cells and cytokines (Chan et al., 2019). Body function decreases with age, including the immune system function. Changes in age-dependent functions contribute to a more harmful effect on adaptive immunity (Schleifer et al., 1989), which is represented by B and T lymphocytes. It has been confirmed that the plasma of MDD patients with non-responsiveness to anti-depressants contains high concentrations of cytokines, such as interleukin 6 (Vogelzangs et al., 2012). Mishra et al. (2018) demonstrated that high concentrations of inflammatory mediators were related to the severity and duration of depressive disorder. Surprisingly, the decreased expression of cytokines and chemokines was found in elderly patients with MDD (Shinko et al., 2020). However, the role of the differential changes of immune response in LOD is poorly understood. The differential changes of immune response may be one of the main factors that make LOD patients different from non-LOD patients.

In recent years, bioinformatics methods have been widely used in different diseases, including LOD (Miyata et al., 2016). A previous study mainly focused on differential genes in the blood of LOD patients, partially revealing the pathological process of LOD (Miyata et al., 2016). However, the ignorance of non-abnormal genes may lead to deviation in the diagnosis of LOD. Different from general MDD, the pathological mechanism of LOD may be more complex. A comprehensive analysis of all genes in LOD may be the key to clarify the mechanism of LOD. The WGCNA can accurately screen diagnostic genes of diseases through the relationship between genes and clinical characteristics of diseases (Langfelder and Horvath, 2008). As a bioinformatics approach commonly used to analyze the changes in the immune function, the CIBERSORT algorithm can reflect the abundance of 22 kinds of immune cells in different populations through the expression of sample genes (Zhou et al., 2021). Therefore, in our study, we used the WGCNA and CIBERSORT algorithm to identify hub genes and immune infiltrating cells in the plasma of LOD patients and explored the potential mechanism, to provide some guidance for the related research on LOD in the future.

## Materials and methods

### Dataset collection

All datasets (GSE76826 and GSE98793) used in this study were downloaded from the Gene Expression Omnibus database. The GSE76826 dataset contains 12 healthy control individuals, 10 patients with LOD (onset age ≥50 years), and 10 patients with LOD syndromal remission. Also, 12 healthy control individuals and 10 patients with LOD were selected for the WGCNA. In addition, 12 healthy control individuals and 10 patients with LOD syndromal remission were selected for the cross-validation of hub genes. The GSE98793 dataset contains 64 patients with MDD, 64 healthy controls, and 64 patients with MDD and generalized anxiety disorder. From the GSE98793 dataset, we selected healthy controls and MDD patients without anxiety disorder for the immune infiltration analysis, whose age ≥50 years. We used the Perl language (version 5.32.1) to convert the probe ID extracted from the downloaded series matrix into a gene symbol. After that, R software (version 3.6.1) was used on the gene expression datasets for normalization and statistics (Ren et al., 2020). We followed the methods of dataset sorting, which were used in a previous literature report (Xu et al., 2022).

### Differentially expressed genes (DEGs) between healthy control individuals and patients with LOD

Compared with the healthy control individuals, the genes with fold changes < −1.4 or >1.4 and an adjusted *p*-value < 0.05 were defined as DEGs. According to the screening criteria for DEGs, we used R software with the “limma” package to complete the screening of DEGs. A heatmap and volcano map were obtained to visualize the top 100 DEGs (50 upregulations and 50 downregulations) through the “pheatmap” package in R software.

### Co-expression network

We used R software with the “WGCNA” package to identify and visualize the co-expressed modules of the genes in the GSE76826 dataset (Langfelder and Horvath, 2008; Xu et al., 2022). The construction of a co-expression network was in accordance with the steps in the previous literature (Xu et al., 2022). First, the sample information on healthy control individuals and 10 patients with LOD in the GSE76826 dataset was matched with the clinical characteristics, followed by clustering to identify outliers. Then, 1–20 were set as the candidate power values. According to the similarity of co-expression, we used the pickSoftThreshold function to calculate the corresponding power values and drew the scatter diagram corresponding to the power values, including the analysis of network topology for various soft thresholds and mean connectivity. Among them, when the R-square of the scale-free topological network fitting model is about 0.9, the corresponding soft threshold is the best power value to establish the adjacency matrix. Next, the TOM matrix was calculated, followed by detecting the modules through the dynamic tree cutting function and merging similar modules according to MEDissThres = 0.25. Finally, all modules had calculated the clinical correlation values and recorded the genes in each module. Only the modules with a correlation index value greater than 0.5 and a *p*-value less than 0.05 were considered the most clinically significant modules for LOD. The gene information in the most clinically significant modules was selected.

### Functional enrichment

We performed functional enrichment analysis on genes in the first two most clinically significant modules, including Gene Ontology (GO) and Kyoto Encyclopedia of Genes and Genomes (KEGG). The functional enrichment results were visualized through corresponding R packages. The criteria for functional enrichment were the *p* threshold <0.05.

### Immune infiltration analysis

To explore the difference in the proportion of immune infiltrating cells (22 kinds in total) in healthy control individuals and patients with LOD, we used the CIBERSORT algorithm to analyze selected samples from the GSE98793 dataset. The results of the immune infiltrating cells were visualized by R software with corresponding R packages.

### Protein–protein interaction (PPI) analysis

Genes in the first two most clinically significant modules were selected to construct a PPI network through the Search Tool for the Retrieval of Interacting Genes (STRING) (Szklarczyk et al., 2011; Szklarczyk et al., 2019). The setting standards of the PPI network are as follows: interaction score >0.7 and isolated nodes removed. We exported the results of the PPI networks as TSV format files, followed by importing into Cytoscape software (version: 3.7.1). The top 10 hub genes in the PPI networks of the selected two modules were calculated by the cytoHubba tool. Finally, the hub genes of selected modules and DEGs were intersected to screen the hub genes, followed by GO, and KEGG analyses.

### Receiver operating characteristic curve (ROC)

We applied the ROC to verify the diagnostic accuracy of the six hub genes in LOD. The value of the area under curve (AUC) of the ROC represented the diagnostic accuracy. We used R software with the “pROC” package to perform ROC analysis and visualize the results. In the GSE76826 dataset, healthy control individuals and 10 patients with LOD were defined as the control group and case group, respectively. According to the respective real expression values of hub genes in the two groups, the ROC curves were drawn using the “pROC” package with R software. Meanwhile, the ROC curves were smoothed using the “smooth” function, and the AUCs of the ROC curves were calculated for each hub gene. AUC values >0.7000 were defined as accurate. For the cross-validation, we re-drew the ROC curve of each hub gene, according to the actual expression values of hub genes in healthy control individuals and patients with LOD syndrome remission to approach the real testing power.

### Least absolute shrinkage and selection operator (LASSO) regression model and correlation between genes and immune cells

According to the expressions of hub genes, we used R software with the “glmnet” package to construct the LASSO regression model. The parameters of the LASSO regression model were set as follows: family = “binomial” and alpha = 1. Tenfold cross-validation was utilized to determine the optimal value of the penalty parameter λ. Next, the genes screened by the LASSO regression model and the genes verified by the ROC were intersected to obtain the final genes. We used “ggpubr” and “limma” packages to visualize the expression level of the final genes. Finally, the correlations between final genes and immune cells were analyzed by Spearman’s method.

## Results

### Research diagram

As shown in Supplementary Figure S1, GSE76826 and GSE98793 datasets were downloaded for this study. We constructed a co-expression network of selected samples from the GSE76826 dataset and identified the hub genes in LOD. To determine the types of immune infiltrating cells of LOD, we used the CIBERSORT algorithm to analyze the selected samples in the GSE98793 dataset.

### DEGs between healthy control individuals and patients with LOD

**FIGURE 1:**
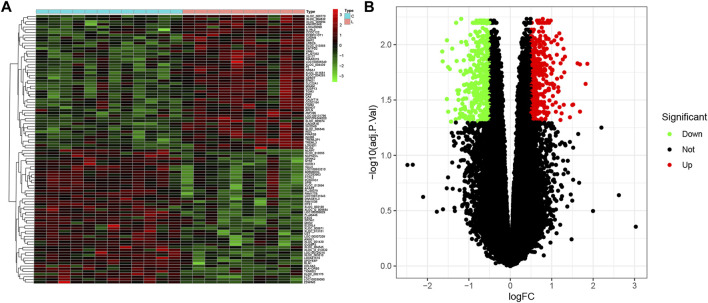
Differential expression analysis. **(A)** Heatmap plot of DEGs of healthy subjects and LOD patients. C, control. LOD, late-onset major depressive disorder. **(B)** Volcano plot of DEGs. Upregulated and downregulated DEGs are shown in red and green, respectively.

According to the defined criteria (fold changes < −1.4 or >1.4 and adjusted *p*-value < 0.05.), we applied R software with the “limma” package to identify the DEGs between healthy control individuals and patients with LOD. A total of 811 DEGs, which consisted of 383 upregulated genes and 428 downregulated genes, were obtained. As shown in Figure 1, we selected the top 50 genes, upregulated or downregulated, respectively, to visualize through a heatmap and volcano plot.

### Co-expression network

**FIGURE 2:**
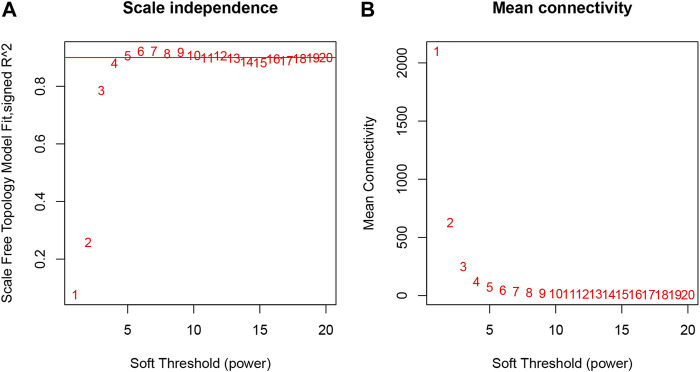
Selection of optimal soft threshold power to construct gene co-expression networks. **(A)** Analysis of network topology for various soft thresholds. The X-axis represents the soft threshold power. The Y-axis represents the scaling topology model fitting index. **(B)** Mean connectivity.

**FIGURE 3:**
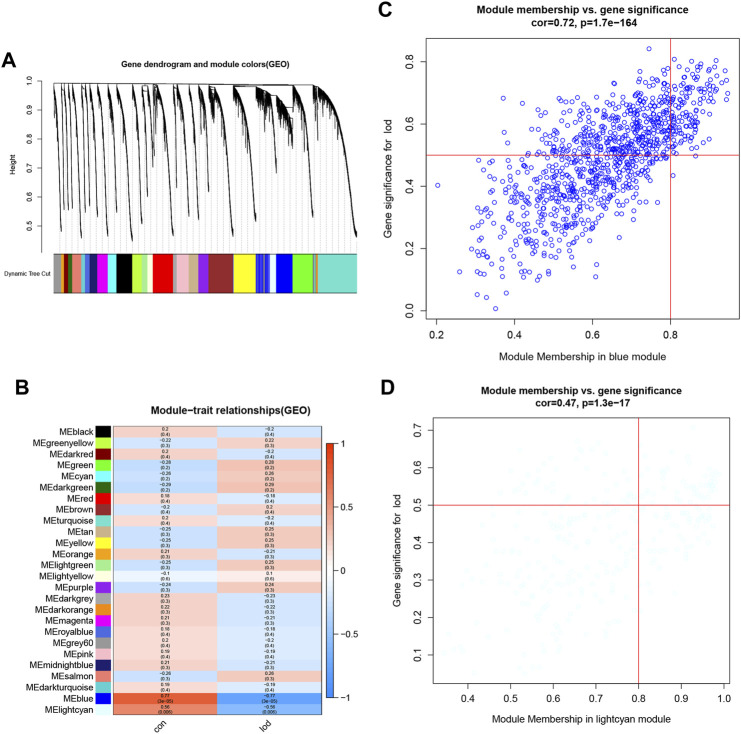
Identification of modules specifically associated with LOD. **(A)** Cluster tree diagram and module feature relation diagram are used to divide modules. **(B)** Heatmap of the correlation between module characteristic genes and different stages. Each cell contains a specific associated exponent and *p*-value. **(C)** Scatter diagram of genes in the blue module. **(D)** Scatter diagram of genes in the light cyan module.

The samples selected from the GSE76826 dataset were used for the WGCNA to explore the hub genes in LOD. The co-expression network was constructed under the soft threshold optimized as 4 (scale-free R^2^ = 0.88), which meets the approximate scale-free topology criterion (Figure 2). A total of 26 modules (Figure 3B) were generated after the combination of similar modules under the criterion that MEDissThres was set as 0.25. In brief, there were 1,025 genes in the blue module, 923 genes in the brown module, 296 genes in the light cyan module, and 1,512 genes in the turquoise module. The specific module information is shown in Table 1.

**TABLE 1:** Gene number information of modules by the WGCNA.

Module name	Number of genes	Module name	Number of genes
Black	597	Light yellow	202
Green–yellow	366	Purple	389
Dark red	160	Dark gray	137
Green	768	Dark orange	65
Cyan	342	Magenta	392
Dark green	154	Royal blue	171
Red	765	Gray	284
Brown	923	Pink	460
Turquoise	1,512	Midnight blue	303
Tan	363	Salmon	346
Yellow	857	Dark turquoise	147
Orange	108	Blue	1,025
Light green	224	Light cyan	295

### The first two most clinically significant modules

As shown in Figure 3, the correlation values and *p-*values between each module and clinical feature are given in detail and visualized. Among them, the blue module had the most significant correlation with LOD (r = −0.77 and *P* = 3e-05). The light cyan module had the second significant correlation with LOD (r = −0.56 and *p* = 0.006) (Figure 3). Therefore, the blue module and the light cyan module were defined as the first two most clinically significant modules. We selected the genes in the first two most clinically significant modules for detailed analysis (Figure 3).

### Functional enrichment analysis of the genes in the first two most clinically significant modules

**FIGURE 4:**
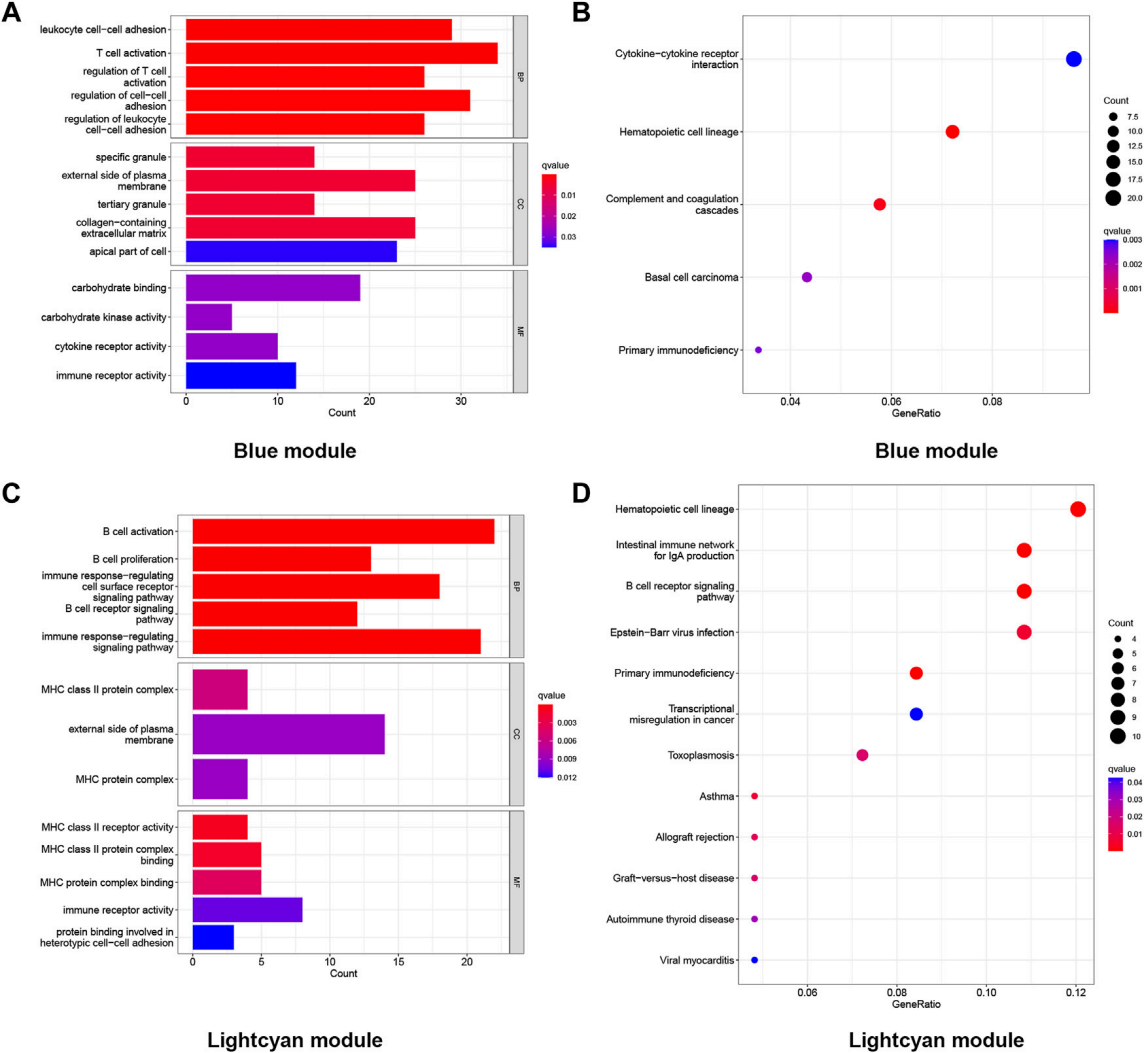
Functional enrichment analysis of the genes in the blue and light cyan modules. **(A)** GO enrichment analysis of genes in the blue module. **(B)** KEGG enriched the regulatory pathways of genes in the blue module. **(C)** GO enrichment analysis of genes in the light cyan module. **(D)** KEGG enriched the regulatory pathways of genes in the light cyan module. Gene ratio, the proportion of genes enriched in this term among all genes used.

Genes in the blue module (*n* = 1,025) and light cyan module (*n* = 296) were selected to perform GO and KEGG enrichment analyses. The results of the GO analysis were divided into the biological process (BP), cellular constituent (CC), and molecular function (MF). As shown in the GO results, in the blue module, the BP of LOD was mainly manifested in T-cell activation, regulation of cell−cell adhesion, and leukocyte cell−cell adhesion. The CC of LOD was mainly manifested in the external side of the plasma membrane, collagen-containing extracellular matrix, and the apical part of cells. The MF of LOD was mainly manifested in carbohydrate binding, immune receptor activity, and cytokine receptor activity. Meanwhile, the results of the KEGG analysis showed that the cytokine−cytokine receptor interaction, hematopoietic cell lineage, and complement and coagulation cascades were the main manifestations. Similarly, as shown in the GO results, in the light cyan module, the BP of LOD was mainly manifested in B-cell activation, the immune response-regulating signaling pathway, and immune response-regulating cell surface receptor signaling pathway. The CC of LOD was mainly manifested in the external side of the plasma membrane, MHC class II protein complex, and MHC protein complex. The MF of LOD was mainly manifested in immune receptor activity, MHC class II protein complex binding, and MHC protein complex binding. In the KEGG analysis of the light cyan module, the hematopoietic cell lineage, intestinal immune network for IgA production, and B-cell receptor signaling pathway were the main manifestations. All functional enrichment results are shown in Figure 4.

### Immune infiltrating cells of LOD

**FIGURE 5:**
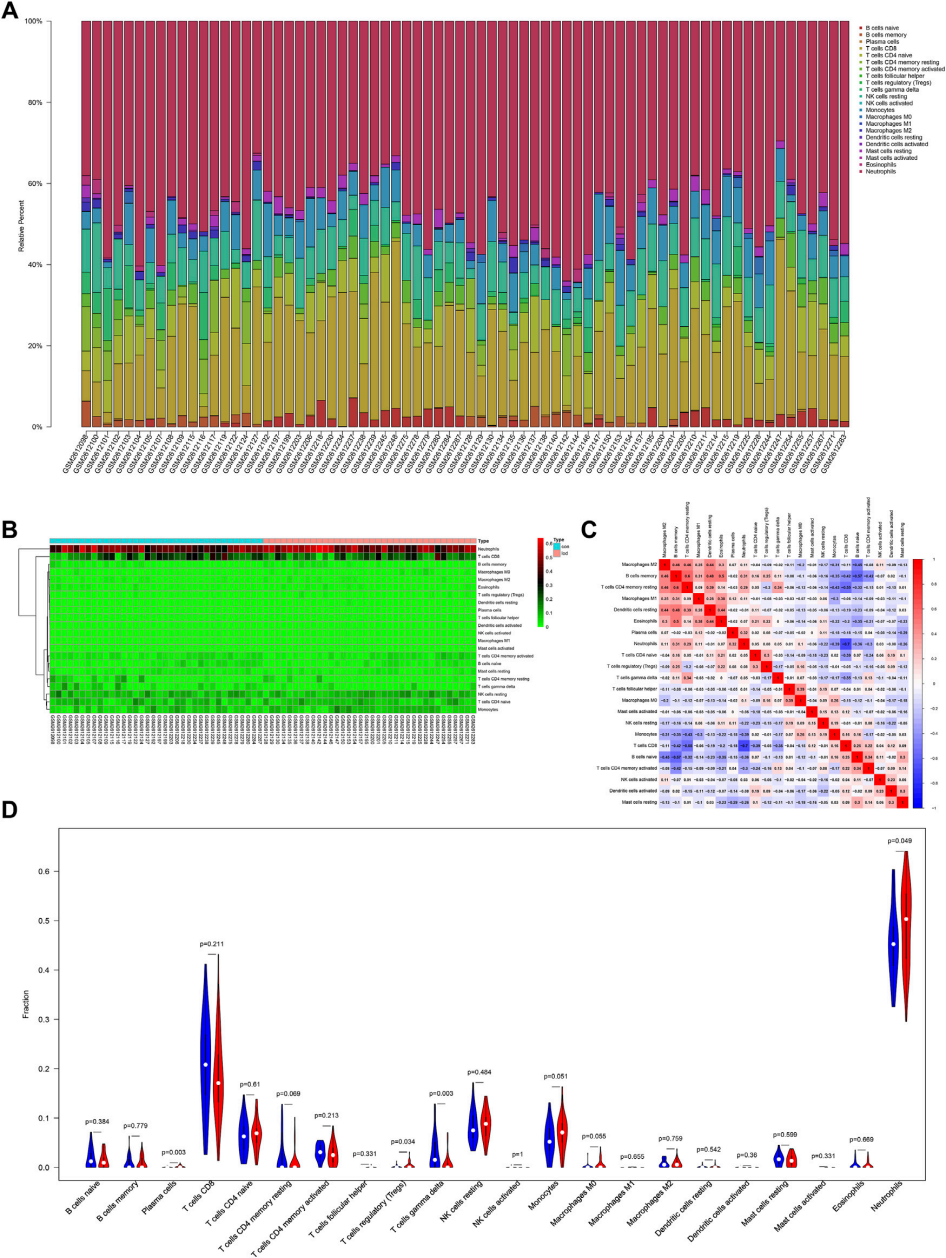
Immune infiltration situation in peripheral blood samples from healthy subjects and LOD patients. **(A)** Histogram of percentage distribution of 22 immune cell subtypes. **(B)** Heatmap of percentage distribution of 22 immune cell subtypes. **(C)** Heatmap of 22 immune cells in both samples. Positive and negative correlations are shown in red and blue, respectively. **(D)** Violin plot of differences in immune cell infiltration between healthy subjects and LOD patients. Blue, healthy subjects; red, LOD patients.

A previous study confirmed that immune function changes play an important role in MDD (Berk et al., 2013). However, the change in the immune function in LOD patients remains unclear. In this study, the results of the functional enrichment analysis of genes in the first two most clinically significant modules showed that the pathological mechanism of LOD involved immune cell response and the regulation of cytokines and chemokines, suggesting immune-related mechanisms may play a role in LOD. To determine the immune infiltrating cells in LOD, we used the CIBERSORT algorithm to identify the immune infiltrating cells of the samples selected from the GSE98793 dataset. The proportion and percentage of 22 subpopulations of immune cells were depicted. It was observed that neutrophils, monocytes, CD8 T cells , and naïve CD4 T cells were the main infiltrating cells. Meanwhile, the results showed that CD4 memory T cells positively correlated with B-cell memory, while CD8 T-cell activation negatively correlated with neutrophils. Compared with healthy controls, LOD patients generally contained a higher proportion of plasma cells (*p* = 0.003), regulatory T cells (Tregs) (*p* = 0.034), and neutrophils (*p* = 0.049), whereas the gamma delta T-cell (*p* = 0.003) fraction was relatively lower. All the results are shown in Figure 5.

### Hub genes of LOD and functional enrichment analysis

**FIGURE 6:**
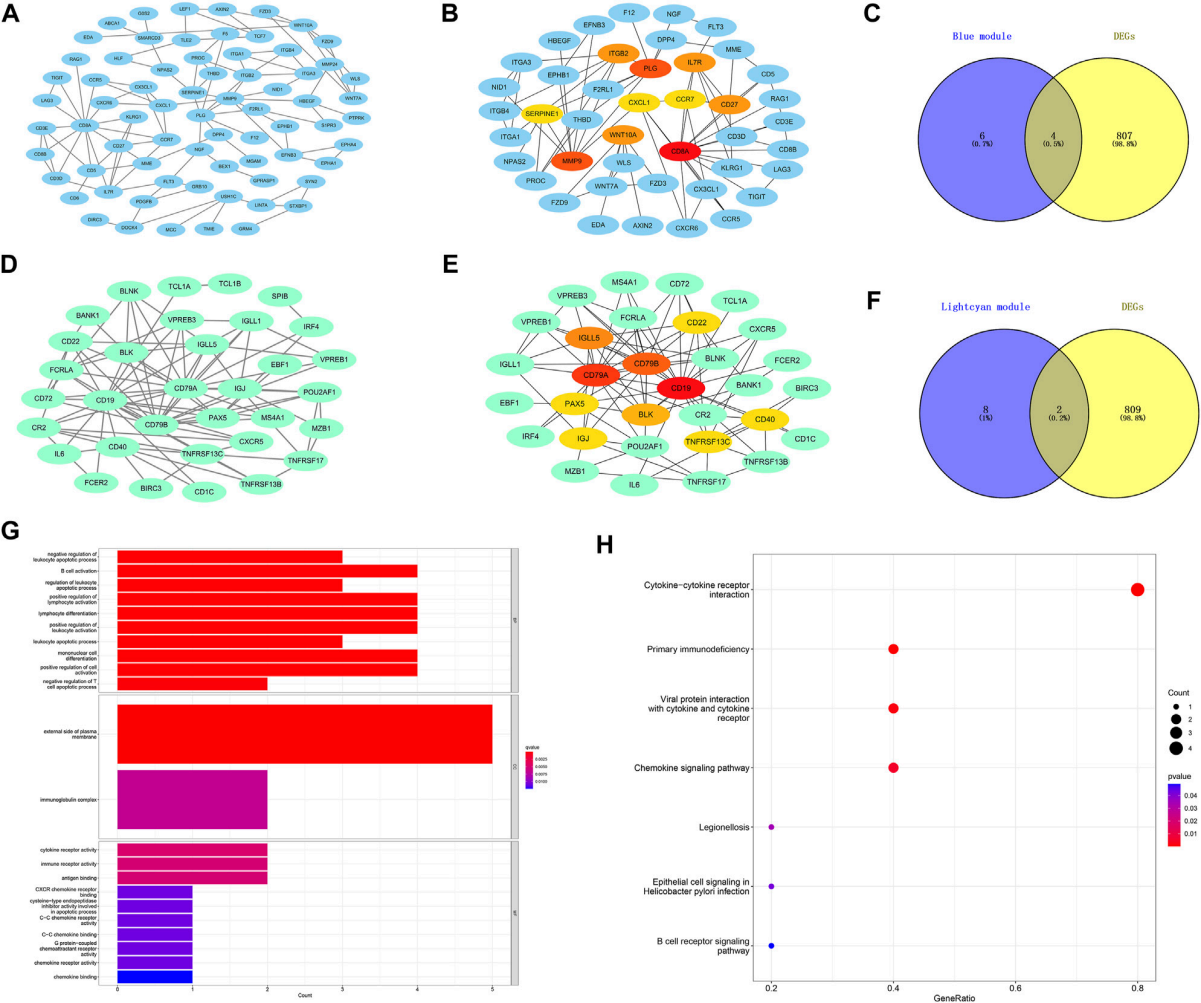
Construction of protein–protein interaction networks with genes in the key modules and functional enrichment analysis of the final hub genes. **(A)** Protein–protein interaction (PPI) networks associated with genes in the blue module. **(B)** Core gene clusters in co-expression networks of the blue module (10 hub genes). **(D)** PPI networks associated with genes in the light cyan module. **(E)** Core gene clusters in co-expression networks of the light cyan module (10 hub genes). **(C,F)** Blue and light cyan modules with the Venn diagrams of DEGs, a total of six final hub genes. **(G)** GO enrichment analysis of the final hub genes. **(H)** KEGG enriched the regulatory pathways of the final hub genes. Gene ratio, the proportion of genes enriched in this term among all genes used.

The PPI networks of the first two most clinically significant modules (blue module and light cyan module) are shown in Figure 6. We used the cytoHubba tool to calculate the top 10 hub genes in the blue module and light cyan module, according to the standard of the node degree. As shown in Figure 6, in the blue module, the top 10 hub genes were *CD8A*, *MMP9*, *PLG*, *IL7R*, *CD27*, *ITGB2*, *WNT10A*, *SERPINE1*, *CXCL1*, and *CCR7*. Meanwhile, in the light cyan module, the top 10 hub genes were *CD19*, *CD79A*, *CD79B*, *IGLL5*, *BLK*, *CD22*, *TNFRSF13C*, *CD40*, *IGJ*, and *PAX5*. The hub genes were screened by intersecting DEGs and the 10 hub genes of the blue module and light cyan module. Finally, the six hub genes were *CD27*, *IL7R*, *CXCL1*, *CCR7*, *IGLL5*, and *CD79A*. In addition, we performed functional enrichment analysis on the six hub genes. As shown in the GO results of the hub genes (Figure 6), the BP was mainly enriched in B-cell activation and positive regulation of lymphocyte activation. The CC was mainly enriched in the external side of the plasma membrane and immunoglobulin complex. The MF was mainly enriched in the cytokine receptor activity, and immune receptor activity. Meanwhile, the KEGG pathway was mainly enriched in the cytokine–cytokine receptor interaction and primary immunodeficiency.

### ROC verification

**FIGURE 7:**
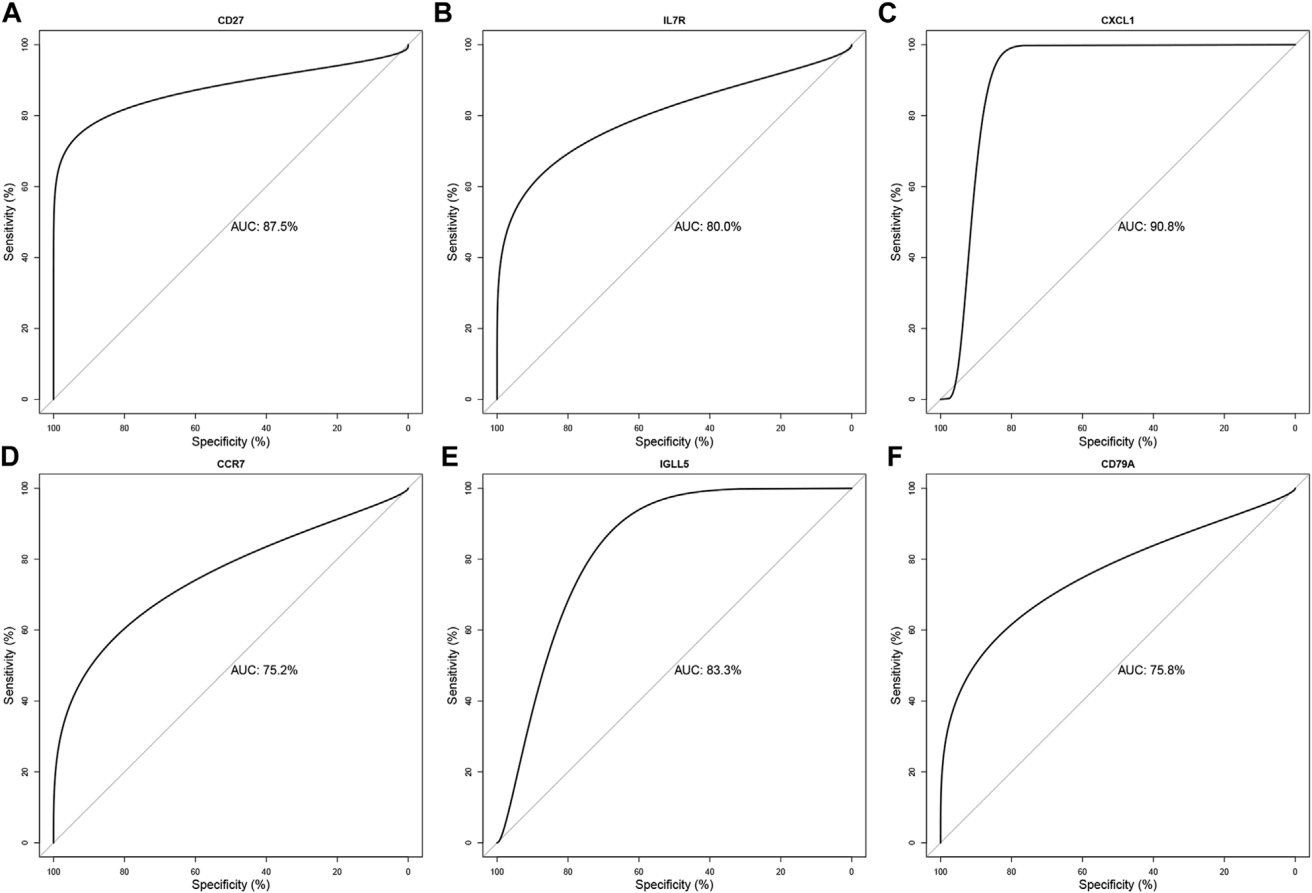
Validation of the diagnostic value for the six final hub genes in LOD patients. ROC curve of *CD27*, *IL7R*, *CXCL1*, *CCR7*, *IGLL5*, and *CD79A* in LOD diagnosis **(A–F)**.

We evaluated the diagnostic accuracy of the six hub genes (*CD27*, *IL7R*, *CXCL1*, *CCR7*, *IGLL5*, and *CD79A*) through ROC verification. As shown in Figure 7, the respective AUC values of the six hub genes were 0.875, 0.800, 0.908, 0.752, 0.833, and 0.758 (Figure 7). All AUC values were greater than 0.700. Meanwhile, ROC results for cross-validation show that the respective AUC values of the six hub genes (*CD27*, *IL7R*, *CXCL1*, *CCR7*, *IGLL5*, and *CD79A*) were 0.809, 0.622, 0.644, 0.417, 0.801, and 0.737 (Supplementary Figure S2). The AUCs of the ROC of *CD27*, *IGLL5*, and *CD79A* were still higher than 0.700. Taken together, our results suggest *CD27*, *IGLL5*, and *CD79A* can be used as the accurate diagnostic indicators of LOD.

### LASSO regression model and correlation between genes and immune cells

**FIGURE 8:**
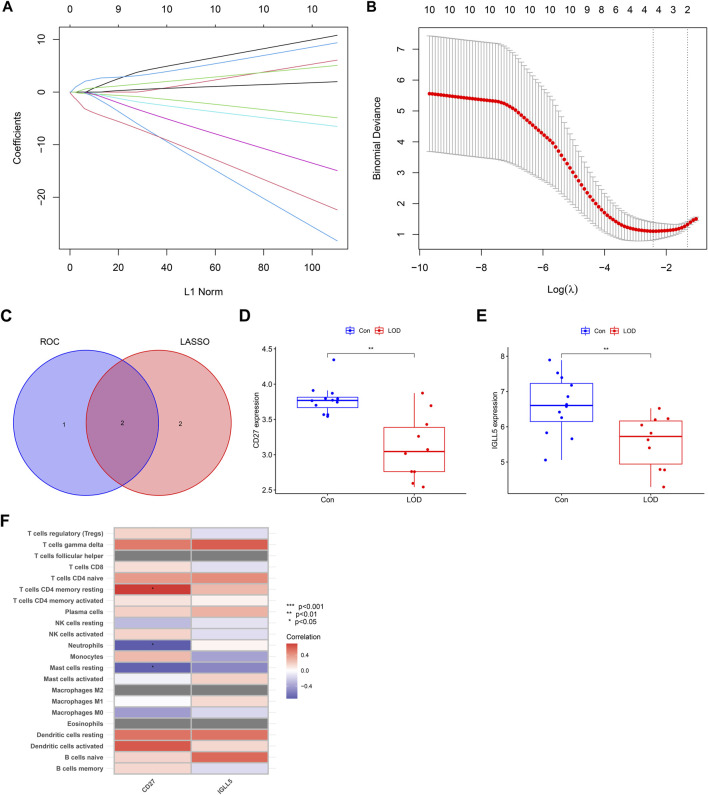
LASSO regression model and correlation between genes and immune cells. **(A)** Ten-fold cross-validation for tuning parameter selection in the LASSO model. **(B)** LASSO coefficient profiles for hub genes of LOD. **(C)** Venn diagram of the genes obtained from LASSO regression and the genes obtained from ROC. **(D,E)** Box plots of the expressions of *CD27* and *IGLL5*. **(F)** Heatmap of correlations between genes (*CD27* and *IGLL5*) and immune cells.

As shown in Figure 8, the error of the LASSO regression model is the smallest when log(λ) is between 2 and 4. Through the LASSO regression model, the obtained hub genes were *IGLL5*, *CD27*, *ITGB2*, and *CXCL1*. Next, we intersected the genes obtained from LASSO regression and the genes obtained from the ROC to obtain *IGLL5* and *CD27*, which had good diagnostic values for LOD. In addition, we found that the expressions of *CD27* and *IGLL5* were significantly decreased in the blood of LOD patients. Furthermore, we found that *CD27* had a significant correlation with CD4 memory resting T cells , neutrophils, and resting mast cells.

## Discussion

MDD is a highly prevalent psychiatric disorder (Sato and Yeh, 2013). MDD is the primary cause of disability, even suicide, which brings heavy pressure and burden to the family and society (Salomon et al., 2012). Growing evidence suggests the patients who had the first onset of MDD at different ages show different depressive symptoms (Naismith et al., 2012; Park et al., 2014). This may explain the different responses to anti-depressants in MDD patients with different ages. In addition, the healthy risk of LOD patients increases greatly with age because of the limitations to diagnosis and the treatment level. Therefore, improving the early clinical diagnosis accuracy of LOD patients is particularly critical for the treatment and protection of patients. We used the WGCNA and CIBERSORT algorithm to identify hub genes and immune infiltrating cells in the plasma of LOD patients. Genes in the first two most clinically significant modules (blue and light cyan modules) to LOD were analyzed for feature and functional enrichment, respectively. Connectedness of PPI network neighborhoods identifies regulatory top 10 hub genes of the blue and light cyan modules. The six hub genes (*CD27*, *IL7R*, *CXCL1*, *CCR7*, *IGLL5*, and *CD79A*) were obtained by overlapping the top 10 hub genes in the two modules with DEGs. The functional enrichment results of six hub genes of LOD were mainly related to the immune function, indicating that the six hub genes may be crucial to the immune process of LOD. Meanwhile, we confirmed the immune infiltrating cells to LOD through the gene expression profiles of the GSE98793 dataset. Finally, we obtained the final genes (*CD27* and *IGLL5*) through cross-validation of ROC and LASSO regression and found that *CD27* was significantly related to the immune correlation in LOD.

It has long been confirmed that inflammation plays an important role in the process of MDD (Berk et al., 2013). The decrease in the body immune function is a kind of physiological change that gradually appears with increasing age. Under the condition of decreased immune function, the inflammatory response in the body is likely to be one of the important promoters of the development of LOD, which may provide an insight into the underlying pathological mechanism of LOD. The immune response process in the body involves many processes, including the infiltration of immune cells and the release of various cytokines and chemokines. PPI networks can provide information on the strength of the relationship between different genes, being used to identify hub genes among multiple genes (Nangraj et al., 2020). In the present study, the results of the functional enrichment analysis with the genes in the first two most clinically significant modules suggest the pathological mechanism of LOD involves the immune cell response and regulation of cytokines and chemokines. The CIBERSORT algorithm confirmed that immune cell infiltration occurred in the blood of LOD patients. In addition, the functional enrichment results of six hub genes (*CD27*, *IL7R*, *CXCL1*, *CCR7*, *IGLL5*, and *CD79A*) of LOD, which were obtained by intersecting the results of DEGs and the PPI network, were mainly related to the immune function. Taken together, these results indicate that the immune response may be crucial to the development of LOD, and six hub genes may be the key to these immune response mechanisms.

CD27 is a member of the TNF receptor superfamily, which is involved in regulating T cells, B cells, and immunoglobulins to mediate the inflammatory response. It has been reported that *CD27* may lead to anxiety or depression in patients with systemic lupus erythematosus by regulating T cells (Gu et al., 2021). As a receptor of interleukin-7 (IL-7), IL7R plays a key role in immune cell development and immune system homeostasis. Previous studies found the notable relevance of IL7R allele polymorphism with the onset age of multiple sclerosis (Salomon et al., 2012; Sato and Yeh, 2013). In addition, recent evidence suggests IL7R, which is associated with lymphoid, regulates the development of tissue-resident macrophages (Park et al., 2014). Recent studies have revealed that the IL7R content in the blood of the elderly is at a low level, which is related to the aging speed and health status of the elderly (Passtoors et al., 2015). In our study, we found the decrease of the IL7R content in LOD, which was in accordance with a previous study (Passtoors et al., 2015).

Chemokines and chemokine receptors play an important role in neurobiological changes in major depressive disorder (Miller and Raison, 2016). It has been demonstrated that the upregulation of the chemokine CXCL1 in the hippocampus participated in depression development (Song et al., 2020). CXCL1 overexpression activates the GSK3β/BDNF/CREB signaling pathway to induce depression-like behavior (Chai et al., 2019). Interestingly, a decline in chemokine gene expression is associated with suicidal behavior among depressive patients. Compared with healthy subjects, patients with suicidal depression had significantly decreased mRNA expressions of chemokines in the prefrontal cortex, including CXCL1 (Pandey et al., 2021). In addition, changed expression of the chemokine CXCL1 was found easily in the plasma of MDD, especially in the elderly (Fanelli et al., 2019). As a homeostatic chemokine receptor, CCR7 was induced by the inflammatory response, playing an important role in neuropsychiatric disorders (Wilson et al., 2009). The central expression of CCR7 proved to help lymph nodes clear memory T cells in the brain to the peripheral nervous system (Charo and Ransohoff, 2006). It has been demonstrated that the neuroprotective effects of CCR7 expression in astrocytes in inflammation during infection (Gomez-Nicola et al., 2010) increased susceptibility to anxiety and depression in CCR7-knockout mice (Harrison et al., 2014). It seems that the decreased expressions of CXCL1 and CCR7 will increase the risk of depression. In this study, low levels of CXCL1 and CCR7 expression were observed in LOD patients, which are in accordance with the results of a previous study (Fanelli et al., 2019).

The *IGLL5* gene encodes one of the immunoglobulin lambda-like polypeptides that is involved in tumor progression. IGLL5 was mainly found to frequently occur in the mutation in chronic lymphoblastic leukemia, lymphoma, and multiple myeloma (Kasar et al., 2015; Pérez-Carretero et al., 2020; Fan et al., 2020; Li et al., 2020; Panea et al., 2019; D'Agostino et al., 2020; White et al., 2018). In the cortex of patients with chronic traumatic encephalopathy, IGLL5 could be a significant diagnosis factor. The *CD79A* gene encodes the Ig-alpha protein of the B-cell antigen component, which is typically associated with the B-cell receptor signaling complex. CD79A was often used as the marker of the B-cell-relevant tumor, such as Hodgkin’s lymphoma (Sakatani et al., 2020). Frequent downregulation or deletion of CD79A could be potential clues for the diagnosis of plasma cell myeloma in the elderly (Tanaka et al., 2009). Previous studies have not reported the association of MDD with IGLL5 and CD79A. In the present study, we confirmed IGLL5 and CD79A in LOD with a low expression level for the first time, which may provide some clues for the future diagnosis and treatment of LOD.

The ROC is widely used to evaluate the diagnostic accuracy of hub genes for diseases based on the characteristics of comprehensive sensitivity and specificity (Zhao et al., 2021). LASSO regression is characterized by variable screening and complexity adjustment by constructing a penalty function while fitting the generalized linear model (Wang et al., 2019). The joint analysis of the ROC and LASSO can further accurately filter hub genes. In our study, we confirmed CD27 and IGLL5 as the accurate predictors of LOD through cross-validation of ROC and LASSO regression models. Meanwhile, the correlation analysis with immune cells showed that CD27 and not IGLL5 had significant correlation with immune infiltrating cells in LOD, indicating that CD27 may be the target of immune function changes in LOD.

## Conclusion

In summary, we explore the difference between healthy subjects and LOD patients by using the WGCNA and immune infiltration. We confirmed the six hub genes and the immune infiltrating cells in LOD. Among the six hub genes, the role of three genes (*CD27*, *CXCL1*, and *CCR7*) in depression has been reported. In addition, there are few studies on IL7R, IGLL5, and CD79A in depression. ROC and LASSO helped us screen CD27 and IGLL5 as the accurate predictors of LOD. In addition, we found that CD27 may be the target of immune function changes in LOD. Our research shows that the changes in immune function may be an important mechanism in the development of LOD. However, there are several limitations to the present study. The detailed mechanism of the final hub genes found in LOD needs to be further studied. In addition, the dominant immune infiltrating cells in LOD need to be confirmed, which may interfere with the development of LOD.

## Data Availability

The datasets presented in this study can be found in online repositories. The names of the repository/repositories and accession number(s) can be found in the article/Supplementary Material.
