# Supplementary material for: Identification of key genes in late-onset major depressive disorder through a co-expression network module
Source: Front Genet. 2022 Dec 6;13:1048761. doi: 10.3389/fgene.2022.1048761 (PMC9763307; doi:10.3389/fgene.2022.1048761)
Supplement: Supplementary file 1 [file Table1.DOCX]

Supplementary Material

# Supplementary Figures


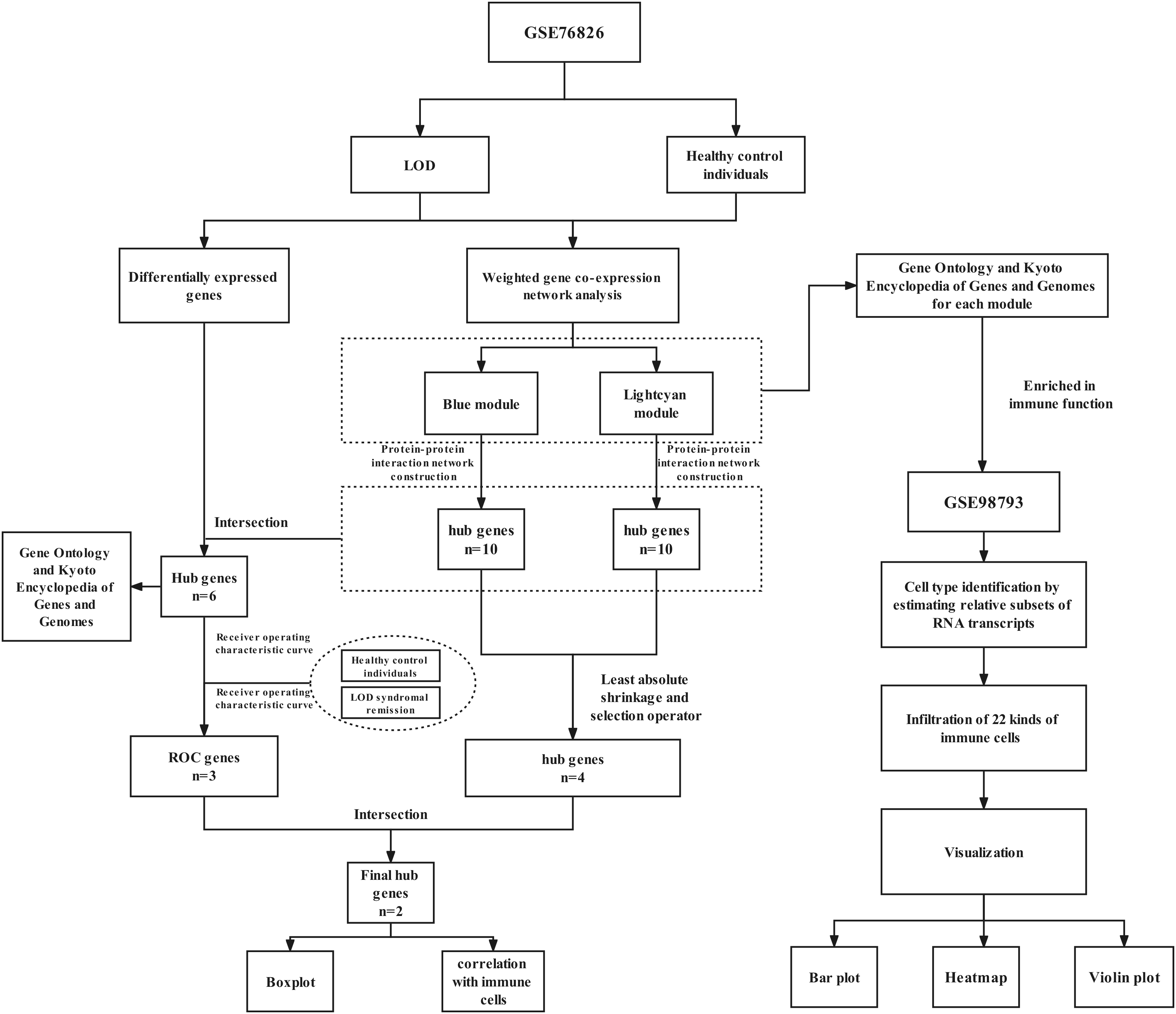


**Supplementary Figure 1.** **Research flow chart**

.


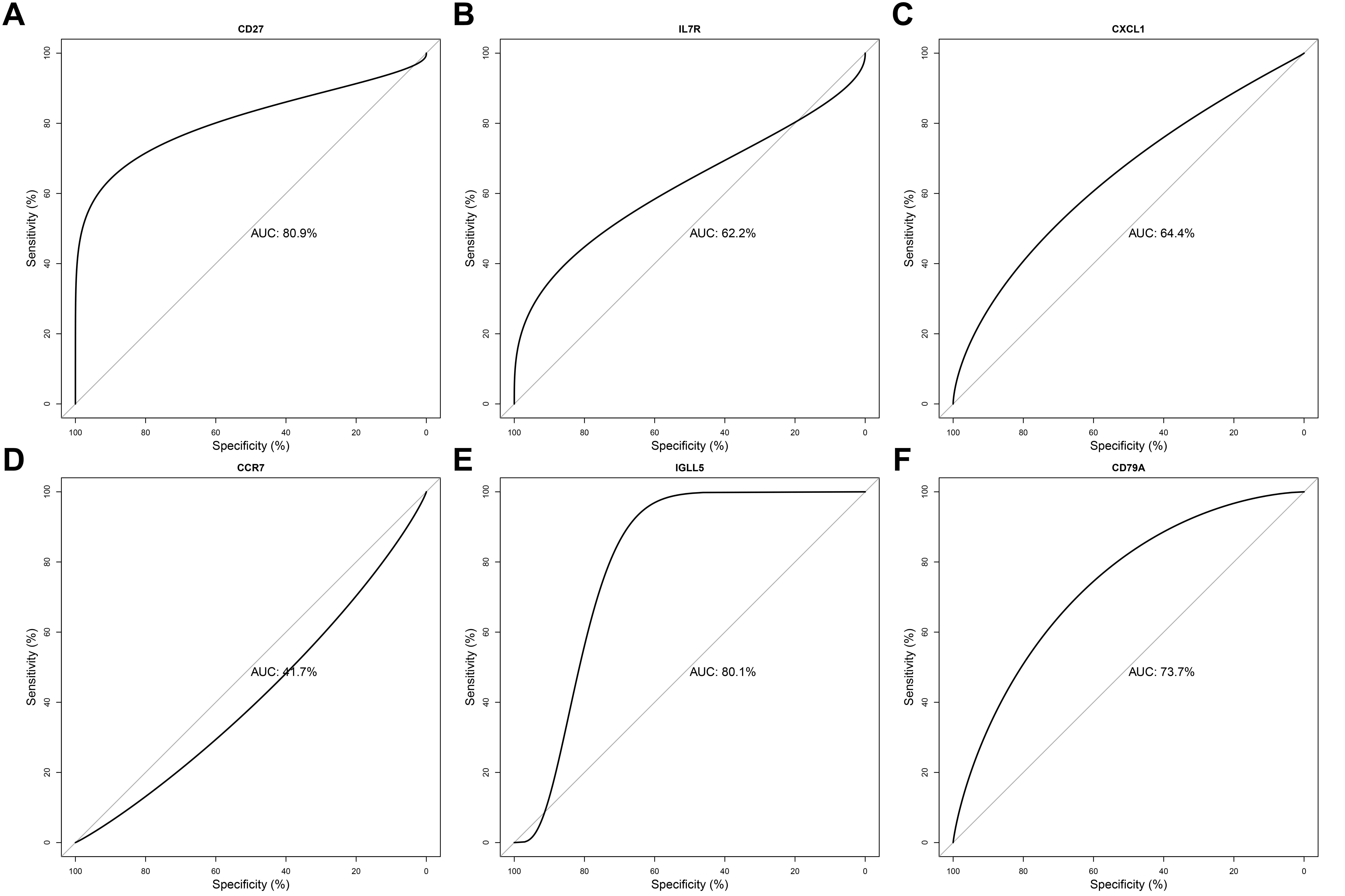


**Supplementary Figure 2 Cross validation of diagnostic value for the six final hub genes.** ROC curve of the CD27, IL7R, CXCL1, CCR7, IGLL5 and CD79A in the LOD diagnosis (A, B, C, D, E, F).
