# Supplementary figures and images for: Identification of key genes in late-onset major depressive disorder through a co-expression network module
Source: Front Genet. 2022 Dec 6;13:1048761. doi: 10.3389/fgene.2022.1048761 (PMC9763307; doi:10.3389/fgene.2022.1048761)

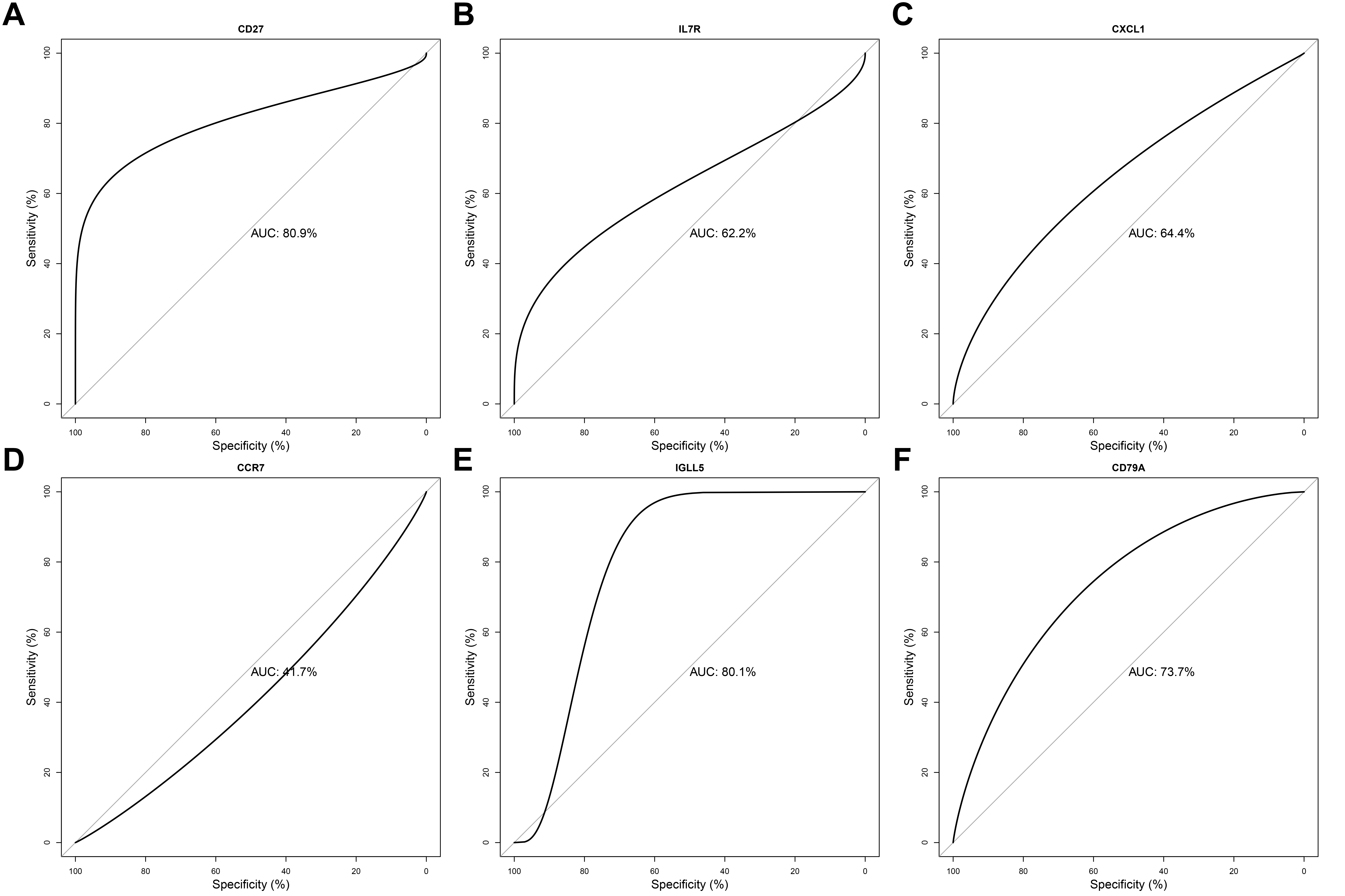

Supplement: Supplementary file 2 [file Image2.TIF]

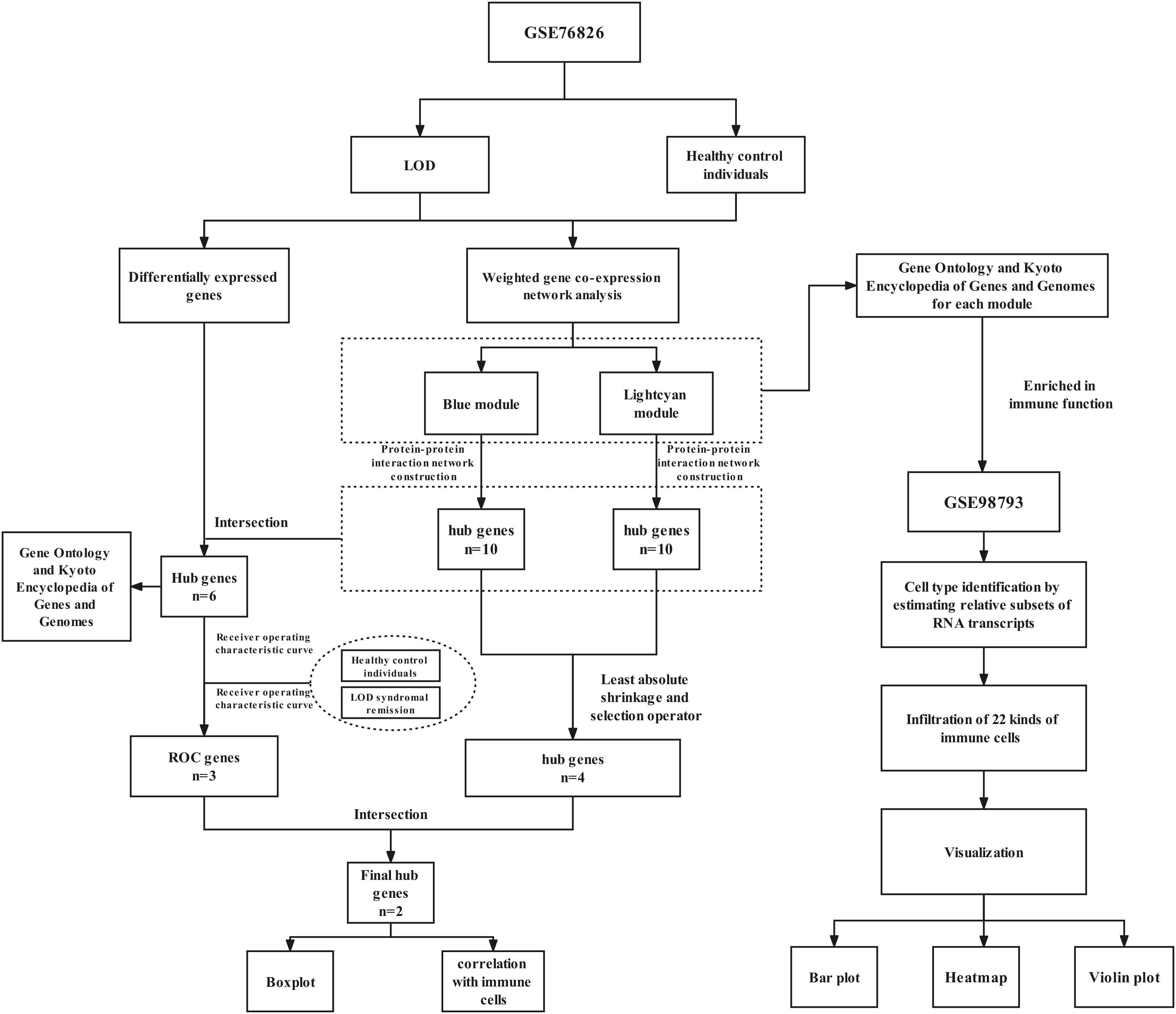

Supplement: Supplementary file 3 [file Image1.TIF]
